# Tracheotomy for Opiate Poisoning

**Published:** 1853-07

**Authors:** 


From the Transylvania Medical Journal.

*Tracheotomy for Opiate Poisoning.*

On the 19th March, at 2 P. M., Mr. Z., of Lexington, about 50 years of age, took a large dose of morphia by mistake. He was found in an hour afterward in profound coma. His breathing was stertorous, and only from two to four per minute. Several physicians were in attendance and employed the usual remedies without effect. On the morning of the 20th, the coma still continued; pupils contracted, and respiration from four to six per minute; pulse 100 and feeble. The operation of Tracheotomy was performed at 11.40, A. M., and the patient survived until 6, A. M., 21st. The operation was suggested by the imperfect character of the respiration, and the increasing cyanosis. The insensibility had become so great that the eyelids and the muscles of the face generally, were quiet under percussion and tickling. In twenty minutes after the operation, the masseter and temporal muscles, and obicularis palpebrarum, contracted when gentle percussion was practised on the temple. In thirty minutes the muscles of the face and neck were generally excitable—pulse a little more full—respiration irregular—from four to sixteen per minute.

Electro-galvanism was employed at 12.41—muscular system very exciteable—respiration averaged about 10 per minute. The cyanic appearance of the surface disappeared, the natural color of the lips and face indicated a good circulation. At the moment when the operation was performed, it was thought impossible for the man to live two hours. The effect of the artificial respiration was to arouse, very sensibly, the activity of the reflux arteries. The brain was too profoundly affected to be relieved.

Would an operation at an earlier hour have produced a better result? The difficult respiration depended partly on the relaxed state of the muscles of the larynx, and the improvement was marked after the introduction of the tube.

Lexington, 9th May. E. L. D.

